# Intravenous iron vs blood for acute post-partum anaemia (IIBAPPA): a prospective randomised trial

**DOI:** 10.1186/s12884-017-1596-x

**Published:** 2017-12-19

**Authors:** Seng Chua, Sarika Gupta, Jennifer Curnow, Beata Gidaszewski, Marjan Khajehei, Hayley Diplock

**Affiliations:** 1 Department of Obstetrics and Gynaecology, Westmead Hospital, Westmead, NSW 2145 Australia; 2 Department of Medicine and Public Health, University of Sydney, Camperdown, NSW 2050 Australia; 3 Department of Maternity and Gynaecology, John Hunter Hospital, Newcastle, NSW 2305 Australia; 4 Department of Haematology, Westmead Hospital, Westmead, NSW 2145 Australia

**Keywords:** Packed cell transfusion, Intravenous iron, Post-partum anaemia

## Abstract

**Background:**

Acute post-partum anaemia can be associated with significant morbidity including a predisposition for postnatal depression. Lack of clear practice guidelines means a number of women are treated with multiple blood transfusions. Intravenous iron has the potential to limit the need for multiple blood transfusions but its role in the post-partum setting is unclear.

**Methods/design:**

IIBAPPA is a multi-centre randomised non-inferiority trial. Women with a primary post-partum haemorrhage (PPH) >1000 mL and resultant haemoglobin (Hb) 5.5-8.0 g/dL after resuscitation with ongoing symptomatic anaemia who are otherwise stable (no active bleeding) are eligible to participate. Patients with sepsis or conditions necessitating rapid Hb restoration are excluded. Eligible participants are randomised to receive a blood transfusion or a single dose of intravenous iron polymaltose calculated using the Ganzoni formula. Primary outcome measures include Hb, Ferritin and C-Reactive Protein levels on Day 7. Secondary outcomes evaluate (i) Hb, Ferritin and CRP levels on Day 14, 28, (ii) anaemia symptoms on Day 0, 7, 14 and 28 using structured health related quality of life questionnaires, (iii) treatment safety by assessing adverse reactions and infection endpoints and (iv) the quantitative impact of anaemia on breast feeding quality using a hospital designed questionnaire.

**Discussion:**

If equivalence in Hb and ferritin levels, symptom scores and safety endpoints is demonstrated, intravenous iron may become the preferred treatment for women with acute post-partum anaemia to minimise transfusion reactions and costs.

**Trial registration:**

Australian and New Zealand Clinical Trials Registry: ACTRN12615001370594 on 16th December, 2015 (prospective approval).

## Background

Post-partum haemorrhage (PPH) commonly complicates pregnancy and can result in acute anaemia [1]. Up to 10% of women with blood loss in excess of 1000 mL will develop severe anaemia (Hb < 8 g/dL), which can be associated with lethargy, decreased mental alertness, physical weakness, poor concentration and a predisposition to post-natal depression [2–4].

Red blood cell (RBC) transfusions have traditionally been used to manage severe anaemia in the pueperium however in the absence of clear transfusion triggers many women without severe symptoms will also receive blood transfusions. This places them at an increased risk of septicaemia, haematological reactions, delayed wound healing and thromboembolism, particularly if they receive multiple transfusions [5]. Blood products are also a scarce and costly resource [5]. Recent institutional guidelines and trial data suggest blood transfusions are likely to be appropriate in patients with Hb < 7 g/dL but not necessarily if alternative therapy is available or if the individual is clinically well compensated [5, 6].

Intravenous (IV) iron therapy has been shown to reduce the requirement for allogenic blood transfusion and can restore haemoglobin levels by an average of 2.5 g/dL at day 5 post infusion with peak effects observed at 3-6 weeks [2, 7–13]. Compared to RBC transfusions, newer iron preparations excluding dextran derivatives are cheap, display a more benign side effect profile than blood transfusions (<0.5% risk of serious adverse events, 1-3% risk of mild adverse events) and have single dosing regimens [14–16].

### Primary objectives

To determine if intravenous iron is non-inferior to RBC transfusion in correcting haemoglobin deficit, replenishing iron stores and improving clinical symptoms in women with acute post-partum anaemia without increasing the rate of adverse outcomes.

### Secondary objectives

To determine if intravenous iron is superior to RBC transfusion in improving the quality of breast-feeding in women with acute post-partum anaemia.

### Hypothesis

Intravenous iron is non-inferior to RBC transfusion in women with acute post partum anaemia in correcting Hb levels and improving clinical symptoms with no increased rate of adverse outcomes.

Intravenous iron is superior to blood transfusion in replenishing pre-pregnancy iron stores.

## Design

This is a multi-centre prospective, randomised, open-label, non-inferiority trial.

### Study settings

Participants will be recruited from at least three Australian hospitals, including Westmead Hospital, a tertiary teaching centre. Secondary sites are eligible if they are willing to participate and have the ability to collect and process blood tests and prepare and administer interventions according to the study protocol in a reasonable timeframe. They must be adequately staffed for recruitment, data collection (including follow-up) and treatment monitoring.

Patients will receive information about the study in antenatal clinics and recruitment will occur post-natally as hospital inpatients

### Participants/eligibility criteria

The sample population will consist of women who sustain a primary post-partum haemorrhage in excess of 1000 mL with a resultant Hb drop of ≥3 g/dL and Hb of 5.5-8.0 g/dL. Asymptomatic women, as well as patients with mild signs and/or symptoms of anaemia including dizziness, increased respiratory rate to >/= 25 on minimal exertion, HR 100-130 or a postural blood pressure drop of >10 mmHg are eligible.

Blood loss will be assessed by weighing all blood stained articles including sponges, sheets, under-pads and clothing. If delivery occurs in the operating theatre (after subtracting liquor volume) suction output will be added. Where possible all blood spillage will be mopped up, weighed and added to the total.

Evidence suggests 90% of women with PPH > 1000 mL have resultant Hb < 10 g/dL thereby defining our haemorrhage cut off [1]. Similarly the nominated minimum Hb is a conservative estimate based on levels used in previous studies evaluating expectant management in patients with acute anaemia [6, 10, 11, 17].

If applicable, enrolment must occur within 48 h of resuscitation and stabilisation. Stabilisation is defined as (1) having no active per vaginal bleeding in excess of expected post-partum losses and (2) having all vital parameters within hospital review criteria for a period of 12 h immediately prior to study recruitment (i.e. all patients having pulse <130 bpm, systolic blood pressure > 90 mmHg, diastolic blood pressure < 110 mmHg, respiration rate < 25, temperature < 38.5 degrees and oxygen saturations >90%).

Patients with significant antenatal or peri-partum complications such as severe pre-eclampsia who are now stable (as per above criteria) are eligible. Women who receive whole blood transfusion, other blood products, antibiotics (other than for sepsis) and intravenous fluid therapy prior to stabilisation will also be eligible.

### Major inclusion criteria

- Age 16-50 years
- Willingness to attend follow up at Day 7, 14, 28

### Major exclusion criteria

- Age < 16 or >50 years
- Refuse consent to blood transfusion or iron infusion
- Co-morbidities necessitating rapid Hb restoration including: cardiac failure, ischemic heart disease, chronic renal failure
- Known hematological malignancy
- Known hemaglobinopathies requiring regular transfusions
- Sepsis (clinical or laboratory evidence—intrapartum fever >38.5 degrees with abnormal vital signs, positive blood culture)
- Contraindications to iron polymaltose (i.e. asthma, known hypersensitivity to iron polymaltose, chronic polyarthritis, acute renal dysfunction, uncontrolled hyperparathyroidism, infectious hepatitis and iron overload (ferritin >1000 ng/L)
- Severe symptoms of anemia including dyspnoea at rest, angina pectoris, syncope or transient ischemic attacks.

### Interventions

All eligible women will be approached and given information about the intervention arms of the study ie. the blood transfusions and intravenous iron including details about the interventions themselves and other scientific and ethical considerations. Those who decline either intervention will be offered oral iron in line with standard practice. These women will be invited to participate in the trial as part of an oral iron subgroup which has an identical follow up protocol. Thse women will be administered 325 mg of oral iron daily. This is not a randomised arm of the study.

Patients in the intervention group will be randomised to receive cross-matched RBC transfusion or an IV iron polymaltose infusion. In patients allocated to RBC transfusion, Hb will be checked after each unit and given to achieve a Hb of at least 9.0 g/dL. Patients who received transfusions will be discharged on 325 mg of oral ferrous sulphate for a minimum of 4 weeks.

Iron poylmaltose was selected instead of iron carboxymaltose as it allows for individualised dose calculations and a larger dose of iron to be administered. Iron carboxymaltose, despite having a shorter infusion time, has a maximum of 1 g that can be administered within a 7 day period.

The dose of iron polymaltose will be calculated according to the Ganzoni formula: Total iron dose (mg) = body weight (kg) x (150-baseline Hb (g/dL)) × 0.24 + 500 mg, assuming a target Hb of 15.0 g/dL which allows for ongoing normal per vaginal loss during the puerperium. For those with Thalassemia minor, the target Hb will be adjusted to reflect the patients pre-pregnancy Hb. Iron will be administered as per hospital protocol which instructs slow infusion at 20-40 mL/h for the first 50 mL, increasing to 120 mL/h based on patient tolerance.

Patients will be pre-medicated with anti-histamine (10 mg cetirizine hydrochloride) and anti-emetic (metoclopramide 10 mg) 1 h prior to the iron infusion to reduce adverse reactions [18]. During the infusion patients will be monitored for adverse affects including itch and urticaria, bronchospasm/dyspnoea, back, joint or muscle pain, nausea, indigestion, abdominal pain, headache, hypotension, tachycardia, syncope, and circulatory collapse [9].

Any patient experiencing an adverse reaction will receive appropriate medical care including additional RBC transfusion if indicated. Infusion rates will be slowed in the instance of mild to moderate reactions and ceased for severe reactions.

A new intravenous cannula will be inserted immediately prior to treatment to minimise the risk of cofounding infection.

### Outcomes

#### Primary outcomes

- Hb, ferritn and CRP levels at Day 7

### Secondary outcomes

- Hb, CRP, ferritin and reticulocyte count at Day 14 and 28
- clinical symptoms at day 7, 14 and 28
- breastfeeding quality at day 7, 14 and 28
- adverse reactions

Quantifying CRP will ensure changes to ferritin levels are not the result of an acute phase response. Reticulocyte count will be used as a marker of bone marrow response [19, 20].

### Statistics

To demonstrate equivalence of the interventions in restoring Hb levels at Day 7 with 90% power at 5% significance (α = 0.025), 116 women need to be recruited to each intervention group. Based on similar studies, this assumes an acceptable difference in Hb of 1 g/dL and an expected difference of 0.7 g/dL [2, 8, 12]. Hence, the final sample size, accounting for a 10% loss to follow up, is a total of 250 women.

Participants experiencing adverse reactions will remain in the trial unless they fail to meet inclusion and exclusion criteria at any point. Data will be analysed according to the intention to treat principle. Primary outcomes and secondary binary outcomes will be analysed using one sided and two group t-tests. The incidence of adverse reactions and symptom scores will be assessed by calculating rates and 95% confidence intervals. Validity of the Post Partum Symptom Scale (PPSS) will be determined at the time of analyses as part of an embedded study and if validated, correlations between subscales and primary outcomes will be analysed.

### Procedures, recruitment and randomisation

Table 1 describes the schedule of enrolment, interventions and assessments during the trial.

**Table 1:** Schedule for enrolment, interventions and assessments

	Enrolment	Allocation	Post Allocation				Close-out
TIMEPOINT	Feb 2016	At Enrolment	0-48 h	Day 7	Day14	Day 28	Day 28
ENROLMENT:							
Eligibility screen	X						
Informed consent	X						
Randomisation		X					
Intervention/Preparation (pharmacy/blood bank)			X				
INTERVENTIONS:							
RBC Transfusion			X				
Intravenous Iron Polymaltose			X				
ASSESSMENTS:							
E-5D,MFI-20) and PPSS symptom scores			X	X	X	X	
Clinical history and examination	X		X	X	X	X	
Hb	X			X	X	X	
Ferritin	X			X	X	X	
UEC	X						
CRP	X			X	X	X	
Reticulocyte Count	X			X	X	X	
Group and hold	X						
Vital Signs		X		X	X	X	

Clinical staff at all sites will be able to contact the research team at any time to enrol participants. Research midwives at the primary site will identify women from daily birth records who have had a PPH >1000mls. These women will be briefed on the study using a structured information sheet about the trial and interventions. Following this they will verbally consent to preliminary bloods including a group and hold, haemoglobin, creatinine (to exclude acute kidney injury), ferritin and CRP. Eligible patients will then provide written informed consent and be enrolled to participate in the study (intervention and follow up phases).

Participants will be stratified as per mode of delivery and randomisation will occur within these groups by a computer generated list, blocked in a 1:1 ratio to each intervention. A central telephone service will be accessed to allocate the intervention and study identification number.

Eligible women who decline partipation in the trial will be offered 325 mg oral ferrous sulphate daily until iron replete, which is in accordance with current standard practice guidelines. This will be mentioned in the consent process.

Blinding during the intervention stage is practically difficult due to strict hospital protocols requiring bedside checks and regulated transparent packaging for all blood products. During the follow up phase however researchers will be blinded to the participant’s intervention and researchers who enrolled a particular participant will not be allocated to conduct their follow up interviews. Participants are reminded not to disclose their intervention to the researcher during follow up. We acknowledge that strict blinding in this phase may be difficult.

### Data collection

Each participant will have a study file labelled with their name, medical record number, study identification number and allocated treatment. This will contain copies of their consent forms (the original will remain in their hospital file while they are an inpatient), questionnaires and comprehensive study checklists template for documenting outcome variables and other clinical data. Files will be kept in locked cabinets on the ward and in the principle researcher’s office after discharge and remain on site for 7 years before being destroyed/archived.

Baseline blood results (Hb, ferritin, CRP) for all enrolled participants will be manually transcribed by researchers from the hospital electronic database into the participant’s study file. Once allocated to an intervention, the number and coding sticker of each blood unit or the serial number and dose of the pre-packaged iron infusion will be recorded in the template.

Patient symptoms will be scored on Day 0 prior to receiving any intervention using previously validated HRQoL questionnaires: the EuroQol-5D (E-5D), the Multidimensional Fatigue Inventory (MFI-20) and our in-hospital devised Post-Partum Symptom Score (PPSS).

The EQ-5D measures a patient’s activity level and emotional attitude at a single point in time, indirectly reflecting symptom severity. It consists of five items (Mobility, Self-Care, Usual Activities, Pain/Discomfort and Anxiety/Depression) where a patient scores: 1 = no problems, 2 = more problems, 3 = extreme problems. The sixth item is a patient’s own global evaluation of their health using a visual analogue scale with a range from 0 (worst imaginable health state) to 100 (best imaginable health state) [21].

The MFI is a 20-item self-report instrument designed to comprehensively measure fatigue. It evaluates: general, physical and mental fatigue and reduced motivation and activity. Patients score each dimension on a scale ranging from 4 to 20 where 4 is optimal. Hence, higher scores indicate more severe symptoms. Scales are balanced to reduce the influence of response tendencies. Each scale sub-total is interpreted independently. Questions are structured to evaluate symptoms over a period of time (5-7 days) which is particularly useful to measure treatment impacts [22]. Both the MFI and EQ-5D have previously been validated in pilot studies of obstetric cohorts [23].

The PPSS developed by clinicians at Westmead Hospital is a 27 point self-report questionnaire measuring frequency across three subscales: frequency of clinical symptoms commonly observed in anaemic patients (6 questions), feeding status indicated by changes to breastfeeding habits (7 questions) and home factors to quantify the amount of assistance received to care for the newborn (11 questions). Validity of the scale to discern a relationship between anaemia severity, treatment and breast feeding quality will be determined at the time of data analyses through an embedded study.

Prior to analysis data will be de-identified using study identification numbers and transferred from study files into an electronic database by a study researcher and re-checked by a second researcher. The database will be on a separate computer at the primary study site, accessible only by study researchers and statisticians who will sign a confidentiality agreement stipulating the terms of their use of the data.

### Follow up

The follow up period consists of 4 weeks with face-to-face visits at Day 7, 14 and 28. At each of these visits women will be seen by a research midwife or doctor. Hb, ferritin and CRP will be checked and results recorded in the study file. Patients will have been discharged with completed pathology forms to facilitate compliance with follow-up laboratory testing. Women will also undergo a clinical examination following a specified checklist to assess for any features of infection or other adverse outcomes . Symptom scores using the three specified scales will be collected at each of the follow up sessions. Research midwives will make all effort to ensure compliance with follow up and can facilitate home visits, including pathology collections if required.

### Monitoring/reporting of data

Given the potential for serious adverse events with each treatment, an independent Data Safety and Monitoring Board has been appointed at the primary study site consisting of a consultant obstetrician, haematologist and anaesthetist who are not directly involved with the study design or execution, as well as an independent research midwife and a statistician. Members will be provided with a charter prior to commencement of the study by study researchers requiring them to convene every 2 months for the duration of the study where they will review the data for completeness, accuracy and to identify incidences and severity of any adverse reactions and their management.

If the total incidence of severe adverse reactions (i.e. anaphylaxis, hematological reaction) exceeds 2 per 50 participants at any time in either treatment group, or if the difference in incidence of serious adverse events is greater than 50%, the study will be suspended until further review. Unscheduled DSMB meetings will be called whenever severe adverse reactions occur in either treatment group.

Conclusions and recommendations regarding study modifications will be presented to the principal researchers after each DSMB meeting and attention to these recommendations will be reviewed at subsequent meetings. Final decisions to terminate the study will be made by study researchers in consultation with the DMSB and the ethics committee.

## Discussion

Moderate to severe acute post-partum anaemia is associated with a number of short and long-term adverse health outcomes hence active treatment is necessary and is commonly indicated by a combination of haemoglobin and iron deficit and symptom severity. Whilst RBC transfusion is the mainstay treatment for severe symptomatic anaemia, wide practice variations exist in the management of mild-moderate anaemia in otherwise clinically stable women. If over treated with blood products, these women are placed at risk of transfusion related adverse effects, which although uncommon, can be life threatening and are almost always associated with ongoing morbidity.

The argument supporting expectant management for these patients has been well outlined in recent studies though there is no standard definition of what expectant management entails. Some institutes advocate the use of oral iron only whilst others prescribe parenteral replacement. Long term data indicates that parenteral iron is faster and more effective than oral iron at restoring haemoglobin and total body iron deficits and has the added advantage of avoiding gastrointestinal side effects which often interrupt compliance in the post-natal cohort [2, 3, 24]. Moreover, the lag period to replenish iron stores and induce erythropoeisis is significantly contracted with IV compared to oral therapy. This, coupled with an attractive safety and tolerance profile as well as being cheaper and more available, introduces parenteral iron as an appropriate alternative to blood transfusions in selected women.

If our study hypotheses are true then intravenous iron presents as a safer, sustained and more cost-effective alternative to blood transfusion in the management of hemodynamically stable women with acute post-partum anaemia. Our findings will be particularly useful for patients ineligible for treatment with blood products due to clinical or religious reasons and in under-resourced settings both within Australia and internationally. Ultimately we would hope these findings support the widespread implementation of parenteral iron into routine post-natal practice policies in an effort to improve patient safety and minimise resource wastage.

## Abbreviations

BP: Blood pressure
CRP: C-Reactive Protein
Hb: Haemoglobin
HR: Heart rate
HRQoL: Health related quality of life
IV: Intravenous
PPH: Primary post-partum haemorrhage
RBC: Red blood cell
RR: Respiratory rate
